# A Polysaccharide Virulence Factor from *Aspergillus fumigatus* Elicits Anti-inflammatory Effects through Induction of Interleukin-1 Receptor Antagonist

**DOI:** 10.1371/journal.ppat.1003936

**Published:** 2014-03-06

**Authors:** Mark S. Gresnigt, Silvia Bozza, Katharina L. Becker, Leo A. B. Joosten, Shahla Abdollahi-Roodsaz, Wim B. van der Berg, Charles A. Dinarello, Mihai G. Netea, Thierry Fontaine, Antonella De Luca, Silvia Moretti, Luigina Romani, Jean-Paul Latge, Frank L. van de Veerdonk

**Affiliations:** 1 Department of Medicine, Radboud University Medical Center, Nijmegen, The Netherlands; 2 Microbiology Section, Department of Experimental Medicine and Biochemical Sciences, University of Perugia, Perugia, Italy; 3 Department of Rheumatology, Radboud University Medical Center, Nijmegen, The Netherlands; 4 Department of Medicine, University of Colorado Denver, Aurora, Colorado, United States of America; 5 Unité des Aspergillus, Institut Pasteur, Paris, France; Washington University School of Medicine, United States of America

## Abstract

The galactosaminogalactan (GAG) is a cell wall component of *Aspergillus fumigatus* that has potent anti-inflammatory effects in mice. However, the mechanisms responsible for the anti-inflammatory property of GAG remain to be elucidated. In the present study we used *in vitro* PBMC stimulation assays to demonstrate, that GAG inhibits proinflammatory T-helper (Th)1 and Th17 cytokine production in human PBMCs by inducing Interleukin-1 receptor antagonist (IL-1Ra), a potent anti-inflammatory cytokine that blocks IL-1 signalling. GAG cannot suppress human T-helper cytokine production in the presence of neutralizing antibodies against IL-1Ra. In a mouse model of invasive aspergillosis, GAG induces IL-1Ra *in vivo*, and the increased susceptibility to invasive aspergillosis in the presence of GAG in wild type mice is not observed in mice deficient for IL-1Ra. Additionally, we demonstrate that the capacity of GAG to induce IL-1Ra could also be used for treatment of inflammatory diseases, as GAG was able to reduce severity of an experimental model of allergic aspergillosis, and in a murine DSS-induced colitis model. In the setting of invasive aspergillosis, GAG has a significant immunomodulatory function by inducing IL-1Ra and notably IL-1Ra knockout mice are completely protected to invasive pulmonary aspergillosis. This opens new treatment strategies that target IL-1Ra in the setting of acute invasive fungal infection. However, the observation that GAG can also protect mice from allergy and colitis makes GAG or a derivative structure of GAG a potential treatment compound for IL-1 driven inflammatory diseases.

## Author Summary

*Aspergillus fumigatus* is an opportunistic pathogenic fungus that primarily causes infections in the immunocompromised host. It is known that *Aspergillus* employs various strategies to evade immune recognition by the host's immune system. Recently, galactosaminogalactan (GAG), a new component of the *Aspergillus* cell wall, was discovered to have potent anti-inflammatory effects in mice making them more susceptible to *Aspergillosis*. In the current study we found that this anti-inflammatory property of GAG was due to its capacity to induce the potent anti-inflammatory cytokine interleukin-1 Receptor antagonist. This cytokine interferes with IL-1 signalling and thereby can reduce IL-1–induced immune responses such as T-cell responses. We also found that the induction of this anti-inflammatory cytokine by GAG correlates with increased fungal burden, and mice deficient for this cytokine were protected against aspergillosis. Additionally, we show that the capacity of GAG to induce the natural regulator of IL-1 signalling could be used in the treatment of IL-1–mediated disease such as allergy and colitis. Our study provides new insights on the immunoregulatory activity of GAG and opens up possibilities to exploit the anti-inflammatory potential of GAG as a therapy for inflammatory diseases.

## Introduction

*Aspergillus fumigatus* is an opportunistic fungus that causes infections under specific conditions, of which secondary immunodeficiency is by far the largest risk factor for the development of invasive infections [1]. In order to initiate an effective host response against *Aspergillus*, recognition of conserved pathogen associated molecular patterns (PAMPs) by specific pattern recognition receptors (PRRs) is required.

*A. fumigatus* has a complex cell wall consisting of polysaccharides that play essential biological functions in fungal cell biology and host-pathogen interactions. Some of these polysaccharides are recognized by various PRRs expressed on human immune cells [2]. However, *A. fumigatus* employs various strategies to evade immune recognition. *Aspergillus* expresses surface molecules that shield PAMPs or can modulate TLR responses [3]. Several surface molecules and PAMPs of *A. fumigatus* have been characterized as being capable of modulating or suppressing the immune response. Rodlets and melanin, that are present on the conidial surface, shield PAMPs that elicit pro-inflammatory host responses [4], [5]. In addition, β-glucan, α-glucan and galactomannan (GM) have been shown to modulate the host immune response [6].

Another cell wall component of *A. fumigatus* that is capable of modulating the immune response is galactosaminogalactan (GAG) [7]. GAG is not expressed on *Aspergillus* conidia, but is exposed when conidia start to germinate and was found to be present in the extracellular matrix that surrounds *Aspergillus* hyphae in aspergilloma isolated from patients and in experimental murine invasive aspergillosis [8]. Furthermore, GAG has been shown to serve as an adhesin of *Aspergillus* [9], [10] and to shield β-glucan moieties on the cell wall [9]. This polysaccharide that is shed into the host environment during *Aspergillus* vegetative growth induces immunosuppressive effects that results in diminished neutrophil recruitment, which predisposes mice to *A. fumigatus* infection [7]. However, the mechanism through which GAG induces immunosuppressive effects as well as its capacity to induce similar immunosuppressive effects on the human immune response were unknown. Therefore, we investigated whether GAG can be immunosuppressive in the human host response against *A. fumigatus*, and we have systematically addressed the possible mechanisms responsible for the anti-inflammatory property of GAG.

## Results

### Galactosaminogalactan modulates human T-helper cytokine responses

**Figure 1:**
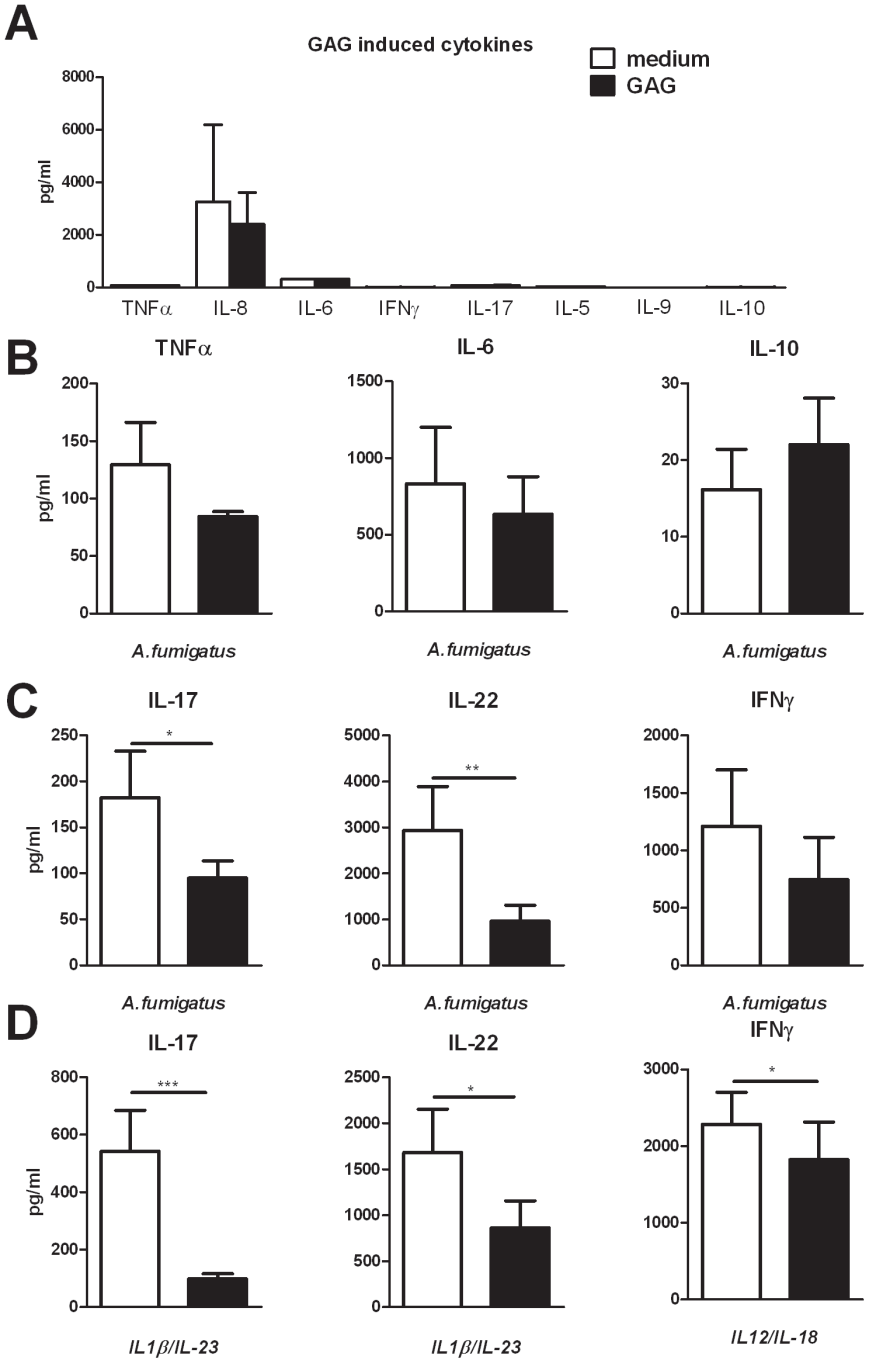
GAG inhibits *Aspergillus*-induced human T-helper cell cytokine production. (A) TNFα, IL-6, IL-8 and IL-10 concentrations in culture supernatants of human PBMCs stimulated for 24 hours with 10 µg/ml GAG and IFN-γ, IL-17, IL-5 and IL-9 concentrations after 7 days of stimulation. (B) TNFα, IL-6 and IL-10 concentrations in culture supernatants of human PBMCs (n = 6 donors) stimulated for 24 hours with heat inactivated *A. fumigatus* conidia (1×10^7^/ml) in the presence or absence of 10 µg/ml GAG. (C,D) IL-17, IL-22 and IFN-γ concentrations in culture supernatants of human PBMCs stimulated for 7 days with heat inactivated *A. fumigatus* conidia (1×10^7^/ml) (n = 10 donors for IL-17 and IL-22, n = 6 donors for IFN-γ) (c), IL-1β/IL-23 (50/100 ng/ml) (n = 14 donors) or IL-12/IL-18 (50/100 ng/ml) (n = 10 donors) in the presence or absence of GAG (10 µg/ml). Data are represented as mean +/− SEM.

To investigate whether GAG can exert immunomodulatory effects in humans, we tested whether GAG induces the production of pro- and/or anti-inflammatory cytokines in human PBMCs. GAG did not induce the proinflammatory cytokines TNFα, IL-6, IL-8, IFN-γ, IL-17, IL-5 and IL-9 (Figure 1A), neither did it induce the anti-inflammatory cytokine IL-10 (Figure 1A). To determine whether GAG modulates *Aspergillus*-induced innate monocyte-derived cytokines, PBMCs were stimulated for 24 hours with *Aspergillus* conidia (these morphotypes of *A. fumigatus* were selected because they do not contain GAG that would interfere with the study of the immunological function of GAG) in the presence or absence of GAG. The presence of GAG did not have a significant effect on the production of the innate cytokines TNFα and IL-6, or the anti-inflammatory cytokine IL-10 (Figure 1B). However, when the production of the characteristic T-helper cytokines IL-17, IL-22 and IFN-γ induced by *A. fumigatus* was investigated, the IL-17 and IL-22 responses were significantly reduced in the presence of GAG (Figure 1C). To determine whether the effects of GAG are specific for *Aspergillus*-driven T-helper (Th) responses, or whether GAG has a general ability to modulate human Th responses, the effects of GAG on cytokine-driven Th responses were studied. GAG significantly decreased the proinflammatory Th cytokine production induced by the cytokine combinations IL-1β/IL-23 and IL-12/IL-18 that induce IL-17/IL-22 and IFN-γ, respectively (Figure 1E). Thus, GAG can inhibit human proinflammatory Th cytokine production induced by *Aspergillus* and cytokine cocktails.

### Galactosaminogalactan induces IL-1 receptor antagonist

**Figure 2:**
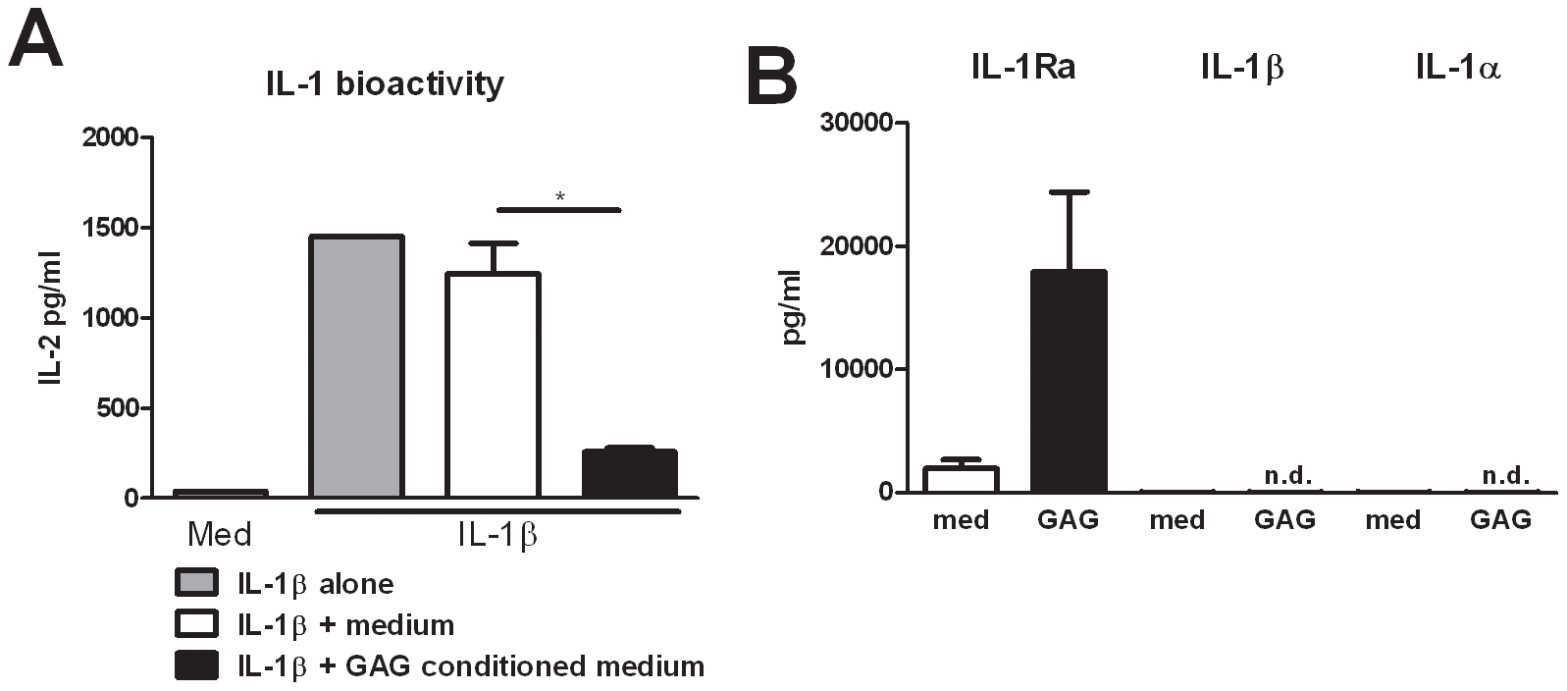
GAG induces interleukin 1 receptor antagonist. (A) IL-1 bioactivity measured as IL-2 production by NOB-1 cells stimulated with 50 ng/ml IL-1β in the presence of culture supernatant of unstimulated PBMCs (medium) or culture supernatants of PBMCs that were exposed to 10 µg/ml GAG for 24 hours (GAG conditioned medium) (n = 6 donors). (B) IL-1Ra, IL-1β and IL-1α concentrations in culture supernatants of human PBMCs stimulated with for 24 hours with 10 µg/ml GAG. Data are represented as mean +/− SEM.

Human Th cytokine responses such as IL-17 and IL-22 production are highly dependent on the IL-1 receptor pathway [11], [12]. To investigate whether the observed modulation of Th cytokines by GAG was due to an interaction of GAG with the IL-1 pathway, we determined the capacity of GAG conditioned medium (culture supernatants of PBMCs that were exposed to 10 µg/ml GAG for 24 hours) to reduce IL-1β bioactivity. Indeed it was shown that GAG significantly reduced the bioactivity of IL-1β while culture supernatants of unstimulated PBMCs did not (Figure 2A). Bioactivity of the IL-1 signalling pathway is dependent on IL-1 receptor agonists (IL-1α and IL-1β) and IL-1 receptor antagonists [13]. One of the natural inhibitors of the IL-1 signalling is the interleukin-1 receptor antagonist (IL-1Ra); therefore the ability of GAG to induce IL-1Ra was tested. IL-1Ra concentrations in the supernatant of the cells stimulated with GAG were significantly increased, whereas GAG induced none of the IL-1 receptor agonists, IL-1α or IL-1β (Figure 2B), showing that GAG has the capacity to modulate immune responses by blocking the IL-1 receptor pathway.

### Suppression of IL-17 and IL-22 by galactosaminogalactan is dependent on IL-1Ra

**Figure 3:**
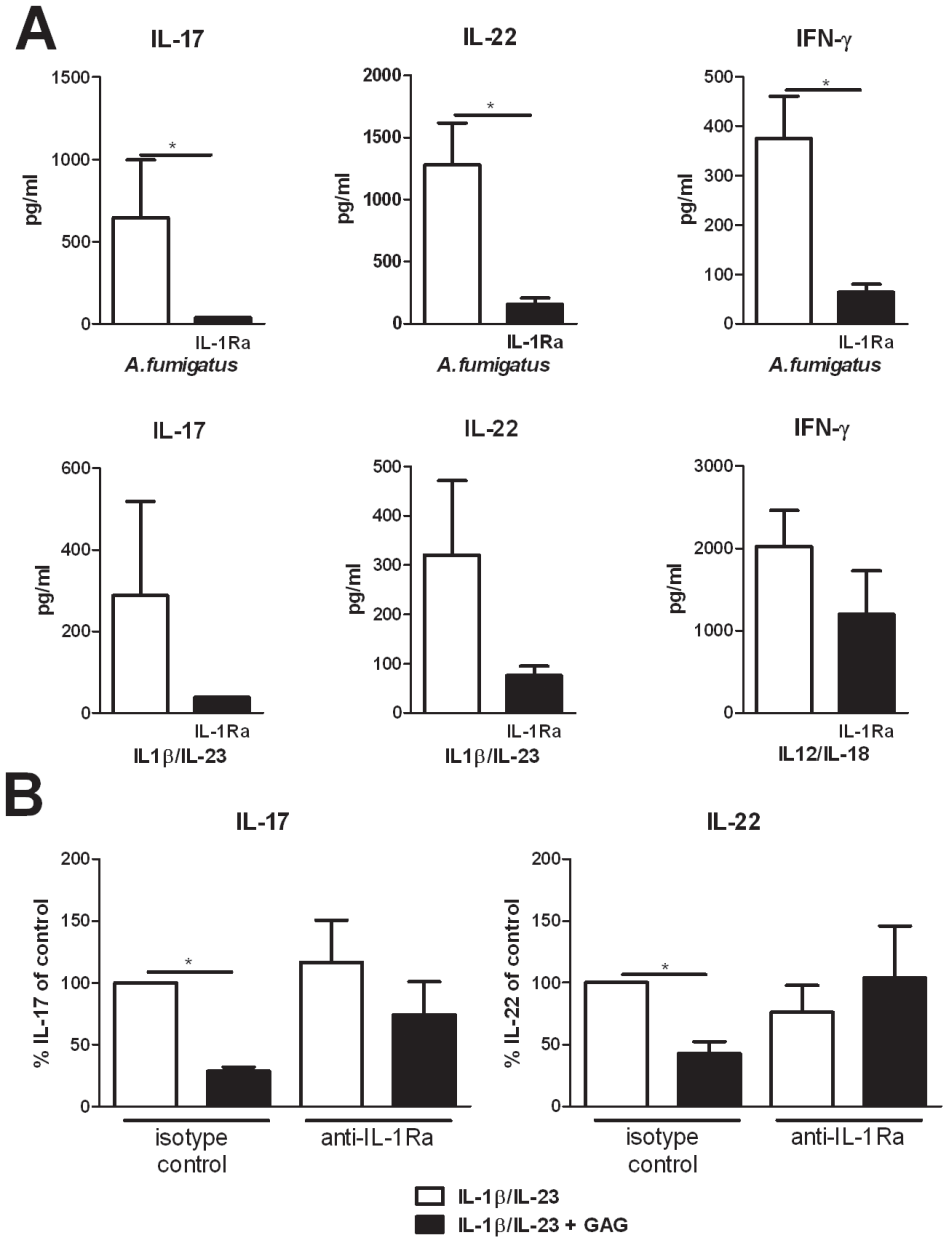
Suppression of IL-17 and IL-22 by GAG is dependent on IL-1Ra. (A) IL-17, IL-22 and IFN-γ concentrations in culture supernatants of PBMCs stimulated for 7 days with heat inactivated *A. fumigatus* conidia 1×10^7^/ml, IL-1β/IL-23 (50/100 ng/ml) or IL-12/IL-18 (50/100 ng/ml) in the presence or absence of recombinant human IL-1Ra (10 ng/ml). Data are represented as mean +/− SEM. (B) Inhibition of IL-1β/IL-23 (50/100 ng/ml) induced IL-17 and IL-22 by GAG (10 µg/ml) in human PBMCs in the presence of isotype control (10 µg/ml) or anti-IL-1Ra (10 µg/ml). The IL-17 and IL-22 production by IL-1β/IL-23 in absence of GAG was set at 100% and mean percentage changes relative to the control are represented +/− SEM.

To demonstrate that IL-17, IL-22, and IFN-γ production by human PBMCs is indeed dependent on the IL-1 receptor pathway and that IL-1Ra can inhibit the production of these Th cytokines, Th1 and Th17 inducing stimuli were studied in the presence or absence of IL-1Ra. Addition of IL-1Ra reduced IL-17, IL-22, and IFN-γ induction by both *Aspergillus* conidia and by IL-1/IL-23 and IL-12/IL-18 cytokine combinations (Figure 3A). To determine whether the immunosuppressive effect of GAG was mediated through the induction of IL-1Ra, PBMCs were stimulated with IL-1β/IL-23 and GAG in the presence or absence of neutralizing anti-IL-1Ra antibodies. GAG reduced IL-17 and IL-22 levels significantly, which was not observed in the presence of neutralizing anti-IL-1Ra antibodies, demonstrating that the inhibitory effects of GAG on Th cytokine production are dependent on IL-1Ra (Figure 3B).

### Galactosaminogalactan induces IL-1Ra in vivo and IL-1Ra increases susceptibility to aspergillosis

**Figure 4:**
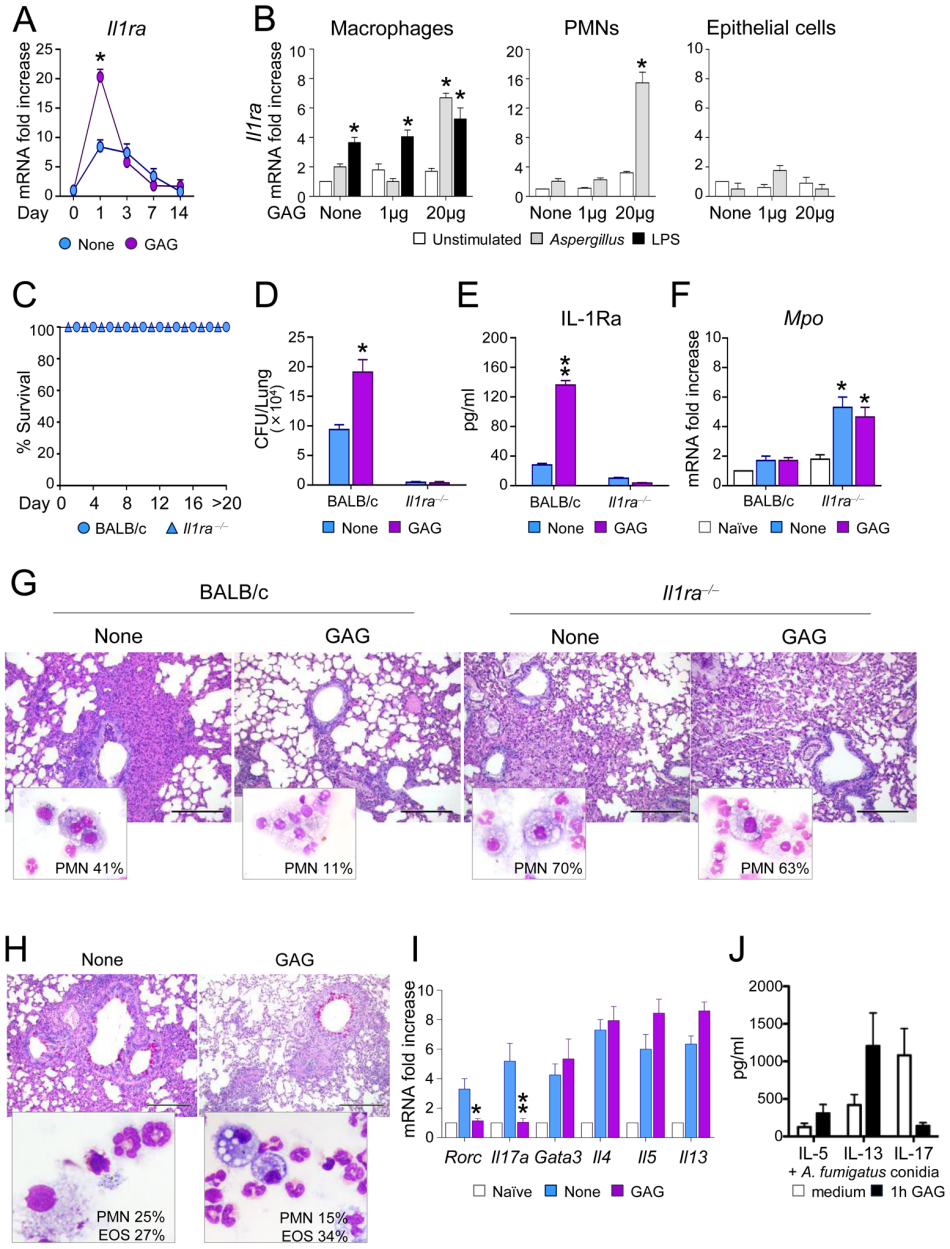
GAG induces IL-1Ra *in vivo* and IL-1Ra increases susceptibility to aspergillosis. BALB/c and *Il1ra^−/−^* mice were intranasally infected with *Aspergillus* conidia and treated with GAG (250 μg/kg intranasally) the day of infection, and 1, 2 and 3 days post-infection. (A) *Il1ra* mRNA expression in lung homogenates of mice with invasive aspergillosis, (B) *Il1ra* mRNA expression in purified cells from lungs of naive mice pre-stimulated with *Aspergillus* conidia or LPS for 1 hour, and exposed to different GAG concentrations for an additional 18 hours. (C) Survival, (D) fungal growth (CFU/lung, mean +/−SEM), (E) protein levels of IL-1Ra, (F) *Mpo* expression in lung homogenates, and (G) BAL morphometry [% polymorphonuclear (PMNs) cells and lung histology (PAS stained sections, bars indicate 20× magnification) of *Aspergillus*-infected mice with or without GAG treatment. Assays were done a day after the last GAG treatment. (H) BAL morphometry [% PMNs or eosinophils (Eo)] and lung histology (PAS stained sections, bars indicate 20× magnification), and (I) expression of Th transcription factors and cytokines in total cells from the draining lymph nodes in mice with ABPA and treated with or without GAG. Naïve means uninfected mice, and none means untreated mice and/or unstimulated cells. (J) IL-5, IL-13 and IL-17 concentrations in culture supernatants of PBMCs pre-incubated 1 h either with medium or GAG (10 μg/ml). After washing, the cells were stimulated for 7 days with heat inactivated 1×10^7^/ml *A. fumigatus* conidia (n = 4 donors). Data are represented as mean +/− SEM. *, p<0.05; **, p<0.01.

The *in vitro* stimulations described above suggest that the immunomodulatory effects of GAG are due to inhibition of IL-1 bioactivity by inducing IL-1Ra. To assess whether this has relevant consequences *in vivo*, we measured IL-1Ra transcription in the lungs of mice infected with *Aspergillus* with or without the administration of GAG. Induction of *Il1ra* was increased in the presence of GAG (Figure 4A). To determine which cells are responsible for the induction of *Il1ra*, we isolated macrophages, neutrophils and epithelial cells from the lungs of naïve mice. Macrophages and neutrophils, but not epithelial cells, expressed *Il1ra* after stimulation with *Aspergillus* in the presence of GAG (Figure 4B). Interestingly, not all microbiological stimuli can prime for increased GAG-induced *Il1ra*, since pre-stimulation with LPS did not increase *Il1ra* induction by GAG (Figure 4B).

To investigate the significance of IL-1Ra *in vivo* and to determine whether the effects induced by GAG are dependent on IL-1Ra, we studied the effects of GAG in wild type (WT) and *Il1ra* ^−/−^ mice with invasive aspergillosis. *Il1ra* ^−/−^ mice were highly resistant to invasive aspergillosis, as indicated by long-term survival (Figure 4C) and reduced fungal burden (Figure 4D). Administration of GAG resulted in increased protein levels of IL-1Ra in the lungs of wild-type mice during infection (Figure 4E). In line with previous observations, GAG increased the susceptibility to invasive aspergillosis in WT mice but not in *Il1ra* ^−/−^ mice (Figure 4D). *Il1ra* ^−/−^ mice had increased *Mpo* expression (Figure 4F) and PMN influx in their respiratory tract (Fig. 4G). As expected, administration of GAG reduced inflammatory PMN recruitment in WT but not in *Il1ra^−/−^* mice (Figure 4G). These data demonstrate that IL-1Ra has an important role in invasive aspergillosis, and support the concept that the induction of IL-1Ra by GAG may have important clinical consequences.

In ABPA, GAG administration decreased PMN recruitment, but not eosinophilic infiltration in the BAL and lung of allergic mice (Figure 4H), a finding consistent with decreased Th17 but not Th2 cell responses in the draining lymph nodes (Figure 4I). To address whether GAG would have similar effects on human Th2 responses, PBMCs isolated form healthy subjects were pre-incubated for 1 h with GAG and subsequently stimulated with *Aspergillus* conidia for 7 days. Similar to mice, IL-17 production decreased in the presence of GAG, but not Th2 cytokines such as IL-5 and IL-13 (Figure 4J). Thus, GAG has the potential to ameliorate Th17-dependent immunopathology in ABPA.

### IL-1Ra induction by Galactosaminogalactan can be exploited as a therapy for IL-1–mediated inflammatory diseases

**Figure 5:**
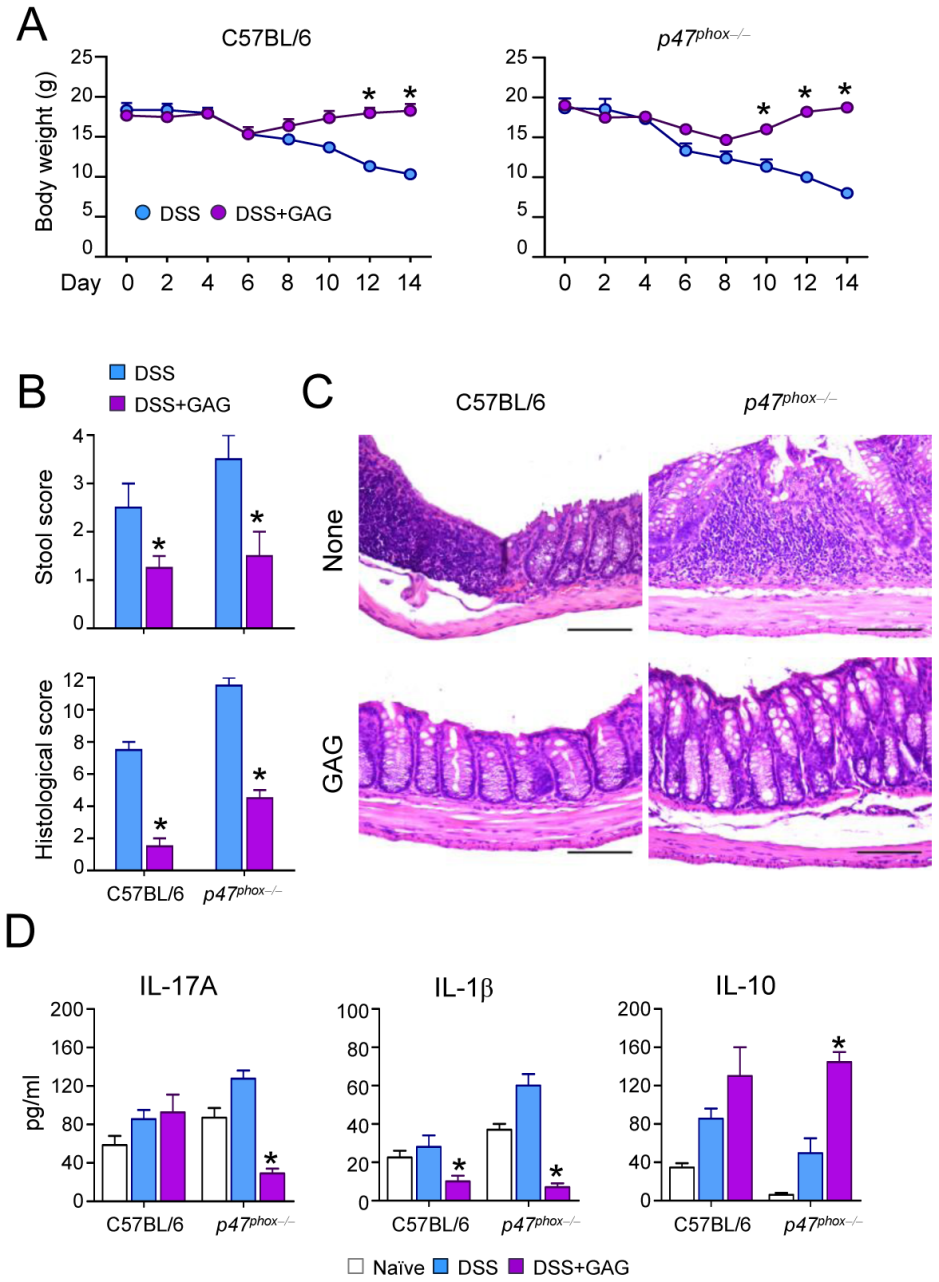
GAG protects mice from experimental DSS-induced colitis. (A) Body weight losses, (B) stool and histological score, (C) histology of colonic sections and (D) cytokine concentrations present in total colonic cells a day after the 7-day of DSS rest in C57BL/6 and *p47^phox−/−^* (CGD) mice with or without GAG treatment. *P<0.05, GAG treated vs. untreated mice.

Since IL-1Ra treatment can be beneficial for autoinflammatory diseases such as chronic granulomatous disease CGD colitis in humans [14], we investigated whether GAG could be beneficial in experimental DSS-induced colitis in mice with chronic granulomatous disease (CGD). The administration of GAG resulted in the amelioration of clinical signs of colitis (weight loss and stool consistency) (Figure 5A-B) and of inflammatory lesions (Figure 5C) in both wild-type and CGD mice. However the protective effects of GAG were most apparent in CGD mice. GAG induced IL-1Ra and, consistently, reduced IL-1β and IL-17 (Figure 5D). Concomitantly, there was an increased production of IL-10, an anti-inflammatory cytokine that plays an important role in the protection of colitis [15], [16]. The effects of GAG on CGD colitis were similar to those of IL-1Ra administration (unpublished data).

## Discussion

In the original report describing GAG [7], it was shown that GAG has anti-inflammatory effects in mice. However, the mechanism through which GAG elicits its immunomodulatory effects remained a question at that time. In the present study, we demonstrate that GAG induces its anti-inflammatory effects by inducing the potent anti-inflammatory cytokine IL-1 receptor antagonist.

IL-1Ra can inhibit the activation of the IL-1 pathway by binding to the IL-1R type 1 receptor and prevents recruitment of the IL-1R accessory protein that is required for signalling. It has been repeatedly shown that IL-1 is an essential proinflammatory cytokine of the innate immunity. A deficient IL-1 pathway is also detrimental for the host, since it is an important protective pathway required to fight infection [17]. Thus the IL-1 axis is a potent pro-inflammatory pathway that needs to be tightly regulated, and IL-1Ra is a crucial player in this regulation. Therefore, it is rather surprising that the role of IL-1Ra in invasive fungal infection has not been studied in detail to date. We observed that the absence of IL-1Ra completely protects mice from developing invasive pulmonary aspergillosis, underscoring the importance of the IL-1 pathway in clearance of an acute invasive *Aspergillus* infection. The observation that GAG induces IL-1Ra *in vivo* identifies GAG as a potent anti-inflammatory molecule that suppresses the IL-1 pathway, subsequently resulting in increased susceptibility to invasive aspergillosis. The relevance of the IL-1 pathway in aspergillosis is underscored by the fact that polymorphisms IL-1 gene cluster polymorphisms are associated with susceptibility to develop in invasive pulmonary aspergillosis [18], and that dectin-1 knockout mice display increased fungal burden and mortality during invasive aspergillosis, which is dependent on IL-1 [19].

One of the major risk factors that increases susceptibility to invasive aspergillosis is neutropenia [20], and neutrophils are crucial for clearing invasive germinating and hyphal forms of *Aspergillus* infection [21]. GAG has been shown to inhibit neutrophil recruitment to the lung, which is at least partly due to neutrophil apoptosis [7]. We observed that in the presence of GAG, IL-1Ra increased during invasive aspergillosis, which correlated with decreased PMN recruitment, and therefore increased fungal burden. In contrast, *Il1ra* ^−/−^ mice displayed increased neutrophil influx when exposed to *Aspergillus*, which could explain the resistance of *Il1ra* ^−/−^ mice to invasive aspergillosis, since they can rapidly and efficiently clear *Aspergillus* conidia due to their increased neutrophil response. In addition to the induction of IL-1Ra by GAG *in vitro* and *in vivo*, we observed that the inhibitory effects of GAG on the proinflammatory Th cytokine response in human PBMCs could be restored in the presence of a neutralizing antibody against human IL-1Ra. Furthermore, the increased susceptibility to invasive aspergillosis induced by GAG is not observed in *Il1ra* ^−/−^ mice. These observations strengthen the hypothesis that the anti-inflammatory properties of GAG are dependent on IL-1Ra.

The anti-inflammatory properties of GAG were present at a concentration of 10 µg/ml, which is a relevant concentration *in vivo*, since *Aspergillus* can secrete GAG in a concentration of 50 µg/ml (data not shown). The finding that antibodies against GAG are present in human serum [8] suggests that there is an adequate exposure of GAG to trigger the immune system. In the present study we were also able to demonstrate that these antibodies do not inhibit the effect of GAG, since we observed significant effects of GAG on IL-1Ra induction and inhibition of IL-17 in the presence of human serum that contained measurable concentrations of antibodies against GAG (data not shown). The relevance of GAG is highlighted by its presence in the extracellular matrix in aspergilloma resected from patients and mice with aspergillosis [8]. It is therefore expected that GAG plays a role in the immunological synapse between host immune cell and the mycelium, not only by inducing anti-inflammatory responses through IL-1Ra but also by shielding β-glucan from recognition, which has been proposed previously [9].

It must be taken into account that in the setting of chronic inflammation in which neutrophils and increased Th17 responses are detrimental for the host, IL-1Ra plays a protective role, due to its significant capability to suppress the IL-1 signaling pathway. This hypothesis is in line with the observation that *Il1ra* ^−/−^ mice develop spontaneous destructive arthritis that is IL-1 and Th17 dependent [22]. The importance of IL-1Ra in controlling IL-1 mediated proinflammatory responses in humans is underlined by a disease called deficiency of IL-1Ra (DIRA). This disease is characterized by the absence of IL-1Ra and severe Th17 mediated responses with neutrophil influx in the skin and bones of these patients, subsequently resulting in severe skin inflammation and osteomyelitis [23]. Therefore, the timing of IL-1Ra induction is of utmost importance to protect the host from infection and overwhelming inflammation. Chronic allergic aspergillosis is associated with excessive inflammation, with increased production of IL-1 and IL-22 [24]. We have demonstrated in the present study that administration of GAG induces IL-1Ra and is able to decrease IL-22 production. Therefore we investigated the effect of GAG in a murine model of allergic bronchopulmonary aspergillosis (ABPA). We observed that the administration of GAG reduces the amount of neutrophils, but not eosinophils in ABPA. Additionally, Th17 responses were downregulated, but not Th2 responses. It is therefore tempting to speculate that administration of GAG can be beneficial in the setting of chronic allergic inflammation that is associated with excessive neutrophil-driven inflammation by reducing Th17 dependent pathology by inhibiting the IL-1 pathway. In addition, GAG also protected CGD mice from experimental colitis. Therefore, we envisage a model in which GAG on the one hand might be detrimental for the host in the setting of an acute infection, and on the other hand could be beneficial for the host during chronic inflammation driven by IL-1. Next to the identification of GAG or IL-1Ra as a therapeutic target for invasive aspergillosis, it is the first time that a polysaccharide produced by a human pathogen has been identified as an inducer of IL-1Ra by cells of the innate immunity without inducing proinflammatory responses, and which has been demonstrated to have therapeutic capacity in IL-1 mediated disease. The search of the sensing and signal transduction cascade activated by this polysaccharide will now be the center of future research.

The data presented here brings new questions into light and opens opportunities for future research. First, one of the most interesting observations is the complete protection of IL-1Ra knockout mice to invasive pulmonary aspergillosis. This opens new treatment strategies that target IL-1Ra in the setting of an acute invasive fungal infection. Second, the significant induction of IL-1Ra by GAG makes GAG or a derivative structure of GAG a potential treatment compound for IL-1–mediated diseases, such as joint, bone and muscle diseases and even very common inflammatory diseases such as diabetes and gout [25]. Previously, we have shown that mitogenic stimulation of monocyte derived macrophages and lymphocytes by αCD3/αCD28 coated beads, or recombinant cytokine-induced IL-17 and IFN-γ production is inhibited in the presence of live *A. fumigatus* [26]. Although these changes in cytokine responses were attributed to changes in tryptophan and kynurenine, it is tempting to speculate that GAG secretion by live *A. fumigatus* could have attributed to the decreased IL-17 production.

In conclusion, our results demonstrate that GAG has potent anti-inflammatory effects in mice and humans that can be explained by the capability of GAG to induce IL-1Ra. These observations help to explain one of the immune-evasive mechanisms of *A. fumigatus*. Moreover, inhibition of GAG or IL-1Ra might prove beneficial in the treatment of acute invasive pulmonary aspergillosis, and GAG might be exploited for treatment of IL-1–mediated inflammatory diseases.

## Materials and Methods

### Ethics statement

All studies with human blood samples were conducted in the Radboud University Nijmegen Medical Centre and the use of healthy volunteers was approved by the institutional ethics review board. Peripheral venous blood samples from healthy volunteers were obtained after written informed consent was provided.

All animal studies were conducted within the University of Perugia and were performed according to the Italian Approved Animal Welfare Assurance A–3143–01. Legislative decree 245/2011-B regarding the animal license was obtained by the Italian Ministry of Health lasting for three years (2011–2014). Infections were performed under avertin anesthesia and all efforts were made to minimize suffering.

### Galactosaminogalactan

Galactosaminogalactan (GAG) was isolated from *A. fumigatus* culture supernatant and purified from the urea-soluble fraction as previously described [7]. Lyophilized GAG was resuspended in 10 mM HCl at 2 mg/ml and used in a final concentration of 10 µg/ml. Before using GAG in stimulation experiments it was incubated with polymixin B to neutralize potential contamination of lipopolysaccharide.

### Stimuli and reagents

A clinical isolate of *Aspergillus fumigatus* V05-27, which has been previously characterized was used for stimulations [27]. Conidia and hyphae were prepared and heat-killed as previously described [6]. A concentration of 1×10^7^/ml was used in the experiments, unless otherwise indicated. Recombinant human IL-1β, IL-23, IL-12 and IL-18 were purchased from R&D Systems (Minneapolis, MN, USA) and were used in end concentrations of 100 ng/ml, 50 ng/ml, 10 ng/ml and 50 ng/ml respectively. Recombinant human (rh) IL-1Ra (Amgen, Inc., Thousand Oaks, CA, USA) was used to antagonize IL-1β signalling at a final concentration of 10 ng/ml. Anti-humanIL-1Ra (R&D) was used to block IL-1Ra in a final concentration of 10 µg/ml, and was compared to isotype control.

### PBMC isolation and stimulation

PBMCs were isolated as described previously [28]. The cells were counted using a particle counter (Beckmann Coulter, Woerden, The Netherlands) and the cell number was adjusted to 5×10^6^/ml. PBMCs were plated in 96-well round-bottom plates (Corning, NY, USA) at a final concentration of 2,5×10^6^/ml and in a total volume of 200 μl. Cells were pre-stimulated for 1 hour with medium or 10 µg/ml GAG. Following prestimulation, the PBMCs were stimulated with culture medium, heat killed *A. fumigatus* conidia (1×10^7^/ml), IL-1β/IL-23 (100 and 50 ng/ml respectively) or IL-12/IL-18 (10 and 50 ng/ml respectively). These experiments were also performed in the presence of 10 μg/ml anti-IL-1Ra antibody or isotype control. Plates were incubated at 37°C, 5% CO_2_ for 24 hours, 48 hours or 7 days. 7 day cultures were supplemented with 10% human serum. In this serum we detected anti-GAG antibodies as described previously [7]. After incubation, culture supernatants were collected and stored at -20°C until cytokine measurements were performed.

### IL-1 bioassay

The murine cell line NOB-1 responds to both human or mouse IL-1 by production of IL-2, furthermore these cells are unresponsive to other cytokines like tumor necrosis factor (TNF), colony stimulating factors (CSFS), IL-3, IL-5, IL-6 and IFN-γ [29]. NOB-1 cells were plated in 96 well flat-bottom plates at a final density of 1×10^6^ cells/ml and were stimulated for 24 hour using culture supernatants of unstimulated PBMCs or PBMCs stimulated in presence of GAG (GAG conditioned medium). After 24 hours of incubation at 37°C, 5% CO_2_ the culture supernatants of the NOB-1 cells were collected and IL-2 production by the NOB-1 cells was measured by ELISA (R&D systems).

### Cytokine measurement

Cytokines were measured using commercially available ELISAs (R&D Systems)(Biolegend, San Diego, CA, USA) (Sanquin, Amsterdam, The Netherlands) according to the protocols supplied by the manufacturer. IL-1α, IL-1β, TNF-α, IL-6, IL-8, IL-1Ra and IL-10 were measured in culture supernatants of 24 hour cultures, and IL-5, IL-9, IL-13, IL-17, IL-22 and IFN-γ were measured in culture supernatants of 7 day cultures.

### Mice

Female, 8- to 10-weeks old, BALB/c (wild-type, WT) mice were purchased from Charles River (Calco, Italy). Breeding pairs of homozygous *Il1ra* ^−/−^ mice on the BALB/c background, were kept under specific-pathogen free conditions at the breeding facilities of the University of Perugia, Perugia, Italy. Experiments were performed according to the Italian Approved Animal Welfare Assurance 229-2011-B.

### Fungal infection, allergy and treatment

Viable conidia from the *A. fumigatus* Af293 strain were obtained as described [30]. For infection mice were anesthetized in a small plastic cage, containing 3% Isofluoran (Isofluran Forene Abbot Scandinavia AB, Solna) before intranasal (i.n.) instillation of a suspension of 2×10^7^ resting conidia/20 µl saline. Mice were treated with 250 μg/kg i.n. of GAG the day of infection and on days 1 to 3 post infection. Mice were monitored for survival, fungal growth (colony forming unit/organ, mean ± SEM), as described [31], histopathology, myeloperoxidase (*Mpo*) and *Il1ra* mRNA expression in lung cells and IL-1Ra production. For histology, sections (3–4 ìm) of paraffin-embedded tissues were stained with periodic acid-Schiff (PAS) reagent. For allergy, mice received an i.p. and s.c. injection of 100 µg of *A. fumigatus* culture filtrate extract (CCFA) dissolved in incomplete Freund's adjuvant (Sigma) followed by two consecutive intranasal injections (a week apart) of 20 µg CCFA. A week after the last intranasal challenge, mice received 10^7^ *Aspergillu*s resting conidia and evaluated a week later (16424201). GAG (250 μg/kg i.n.) or vehicle alone was administered daily, for a week, in concomitance with the *Aspergillus* infection.

### Collection of broncho-alveolar lavage (BAL) fluid

Lungs were filled thoroughly with 1 ml aliquots of pyrogen-free saline through a 22-gauge bead-tipped feeding needle introduced into the trachea. The lavage fluid was collected in a plastic tube on ice and centrifuged at 400 g, 4°C, for 5 min. For differential BAL cell counts, cytospin preparations were made and stained with May- Grünwald Giemsa reagents (Sigma-Aldrich). At least 200 cells per cytospin preparation were counted and the absolute number of each cell type was calculated. Photographs were observed using a BX51 microscope (Olympus, Milan, Italy) and images were captured using a high-resolution DP71 camera (Olympus).

### Dextran sulfate sodium–induced colitis

Mice received either regular drinking water (control) or 2.5% dextran sulfate sodium (DSS) in drinking water for 7 days and then allowed to recover by drinking water alone for an additional 7 days. GAG was given intraperitoneally (1 mg/kg) daily for a week. Weight changes were recorded daily, and the day after the 7-days of rest mice were killed and tissues were collected for histology and cytokine analysis. Colonic sections were stained with H&E [32]. To assess colitis severity, stool and histological scores were used that recently were introduced and proven sensitive to experimental therapy [33].

### Cell purification and cell cultures

Purified peritoneal CD11b^+^ Gr-1^+^ polymorphonuclear neutrophils (PMNs) (>98% pure on FACS analysis) were obtained as described [34]. Lung epithelial cells were isolated as described [35] Murine macrophages were isolated from total lung cells after 2 hours plastic adherence at 37°C. PMNs, epithelial cells and macrophages were exposed to unopsonized *Aspergillus* conidia at the ratio of 1∶1 or LPS (10 ng/ml) at 37°C for 1 hour in the presence of different concentrations (1 or 20 µg/ml) of GAG for 18 hours before the assessment of *Il1ra* mRNA expression.

### Statistical analysis

The differences between the various stimulations were analyzed with the Wilcoxon signed rank test (p-value of <0.05 was considered statistically significant). All experiments were performed at least twice and data represent cumulative results of all experiments performed and are presented as mean +/− standard error of the mean (SEM) unless otherwise indicated. Data was analyzed using GraphPad Prism v5.0.
